# Prevalence and characteristics of tobacco use among adults in Kazakhstan: A cross-sectional National Survey

**DOI:** 10.1016/j.gloepi.2025.100194

**Published:** 2025-03-06

**Authors:** Anel Ibrayeva, Marat Shoranov, Rassulbek Aipov, Adil Katarbayev, Shynar Tanabayeva, Ildar Fakhradiyev

**Affiliations:** a S.D. Asfendiyarov Kazakh National Medical University, Almaty, Kazakhstan; b College of Medicine, Korea University, Seoul, South Korea

**Keywords:** Smoking prevalence, Tobacco use, Public health, Smoking cessation, Kazakhstan, Tobacco control policies

## Abstract

**Background:**

Smoking remains a major public health concern worldwide, contributing significantly to morbidity and mortality. Despite the implementation of tobacco control measures, smoking prevalence in Kazakhstan remains high. This study aims to assess the prevalence, demographic characteristics, and regional variations in smoking habits among adults in Kazakhstan.

**Methods:**

A cross-sectional national survey was conducted from October 2021 to May 2022, covering all 17 regions of Kazakhstan. A total of 6720 adults aged 18–69 years participated, selected using a weighted multistage cluster sampling method. Data were collected through structured interviews based on the WHO STEPwise approach. Smoking status, tobacco consumption patterns, and smoking cessation attempts were analyzed. The results were reported as means with 95 % confidence intervals (CI).

**Findings:**

The overall smoking prevalence was 19.1 %. Smoking was significantly more common among men (30.3 %) than women (7.9 %). The highest smoking prevalence was observed in the 30–44 age group (44.2 %) and among private-sector employees (53.2 %). Regional differences were notable, with the lowest smoking prevalence in Atyrau (9.2 %) and the highest in Pavlodar (30.4 %). Among current smokers, 89.1 % smoked daily, with an average of 11.8 cigarettes per day (95 % CI: 11.4–12.2). Only 36.7 % of smokers who visited healthcare professionals in the past year received advice to quit. Additionally, 42.8 % attempted to quit smoking in the past 12 months. Passive smoking exposure was common, with 26.8 % of women and 22.3 % of men exposed at home, and 30.2 % of men and 14.4 % of women exposed at work. The prevalence of smokeless tobacco use was low (1 %).

**Interpretation:**

Smoking remains prevalent among adults in Kazakhstan, with significant differences by gender, age, occupation, and region. The high prevalence of daily smoking and the low frequency of smoking cessation advice from healthcare professionals indicate the need for stronger tobacco control policies, targeted public health campaigns, and enhanced smoking cessation support programs. These findings provide a basis for future research and policy-making efforts aimed at reducing tobacco consumption and its associated health risks.

## Introduction

In 2020 alone, smoking tobacco products caused 7 million deaths [1], making it a significant contributor to mortality worldwide. Smoking is a leading cause of preventable diseases such as lung cancer, cardiovascular disease, and chronic respiratory disease [2]. These health implications not only lead to human suffering but also impose a significant economic burden, with the annual costs associated with tobacco use surpassing US$1 trillion [3].

In 2019, the global prevalence of smoking was 32.7 % among men and 6.6 % among women. However, these rates vary significantly from country to country. For instance, smoking prevalence ranged from 7.3 % in Peru to 64.6 % in Timor-Leste, illustrating the diverse tobacco consumption patterns across different regions [4]. In Europe, the overall smoking prevalence was 26.3 %, with substantial disparities between countries: Sweden had the lowest prevalence at 7.2 %, while Greece had the highest at 36.6 % [5].

While smoking rates have been declining in low- and middle-income countries over the past two decades, projections indicate that smoking prevalence will remain significant, with middle-income countries expected to have a prevalence of 20 % and low-income countries at 12 % [6]. Currently, Kazakhstan, according to the World Bank classification, is included among middle-income countries [7].

Kazakhstan is the largest country in Central Asia. In 2006, it signed the WHO Framework Convention on Tobacco Control (WHO FCTC). Since then, the country has worked to strengthen its legislation to combat tobacco smoking. Starting in 2009, the Government of Kazakhstan adopted several important tobacco control measures: a minimum age of sale for tobacco products, bans on tobacco use in certain public places, and standardized health warning images on cigarette packages. Through the Tax Code, the excise tax on tobacco products was raised annually from 19 % to 30 % between 2015 and 2019 [8,9].

Despite these measures, the prevalence of smoking in Kazakhstan remains high. According to the Global Adult Tobacco Survey (GATS) conducted in 2014, there has been a lack of continuous monitoring or detailed analysis of smoking and other tobacco use in Kazakhstan since 2019, including an examination of demographic variations and smoking patterns.

While GATS 2019 provided key data on tobacco use, certain aspects were not thoroughly examined, highlighting the critical relevance and necessity of this study. Specifically, GATS 2019 offered national-level data but did not explore smoking across different regions and demographic groups, nor did it consider the socio-cultural factors influencing smoking behavior. The survey lacked a detailed assessment of factors such as residence, education, occupation, marital status, BMI, blood pressure, and heart rate, all of which are studied here with regard to the respondents' gender. These factors are essential for developing targeted public health interventions.

Additionally, a 2021 study examined smoking prevalence in the Zhambyl region with a sample of 1201 individuals. However, these data, drawn from just one of Kazakhstan's 17 regions, cannot accurately represent overall tobacco use in the country [12]. Each region has unique demographic, economic, and socio-cultural characteristics that affect smoking behaviors among its population.

Our research aims to address these gaps by analyzing the latest data on smoking prevalence across Kazakhstan, with a specific focus on demographic variations in smoking habits. This paper offers a thorough view of the current issues and lays the groundwork for future policy decisions in public health management.

The purpose of the study was to conduct a detailed assessment of smoking prevalence among the adult population of Kazakhstan and to identify variations and differences in smoking behavior across various demographic groups.

## Methods

### Ethical considerations

The Local Ethics Committee of the S.D. Asfendiyarov Kazakh National Medical University approved this study (Protocol No. 12 (118), September 28, 2021). Additionally, it was registered on ClinicalTrials.gov under the identifier NCT05122832. All methods were performed in accordance with relevant guidelines. Verbal informed consent was obtained from all study participants. For illiterate participants, informed consent was obtained from their guardian or legally authorized representative. The study was conducted in accordance with the Declaration of Helsinki.

### Study design

Our study employed a cross-sectional design, focusing on the adult population of Kazakhstan from October 1, 2021, to May 30, 2022. A total of 6720 participants (50.1 % male, 49.9 % female) were voluntarily recruited from all 17 regions of the country, including the cities of Almaty, Astana, and Shymkent. The sample mirrors the national census data (48.7 % male and 51.3 % female). The average age of respondents was 40 ± 14 years for males and 41 ± 14 years for females, similar to the national average age of 39.6 years (excluding children under 17). In terms of ethnicity, 65.1 % of respondents identified as Kazakh, aligning with the national figure of 70.2 % (stat.gov.kz). These comparisons demonstrate that our sample is largely representative of Kazakhstan's adult population, albeit slightly older due to the focus on individuals aged 18 and above.

### Study context

Kazakhstan, located in Central Asia, is administratively subdivided into 14 regions, three cities of republican significance (Astana, Almaty, and Shymkent), and 177 districts. The majority of the country's 20 million inhabitants live in urban areas, despite its low population density of 6 people per square kilometer.

### Sampling design

The target population was adults aged 18–69 years. We used a weighted multistage cluster sampling method, segregating participants into four age brackets: 18–29, 30–44, 45–59, and 60–69. The study sample size was determined using the WHO STEPS sample size calculator (Excel format), applying the following:

Hypothesized prevalence of risk factors at 0.5;
Standard error of 0.05;
Design effect coefficient of 1.5;
Projected response rate at 70 %.
The resulting required sample size was *n* = 6585.
Sampling steps:

(1) Selection of the primary sampling units (PSU) – districts and cities

PSUs (clusters) were proportionally selected across all economic regions of Kazakhstan. Information about districts and cities was obtained from the Agency for Strategic Planning and Reforms of the Republic of Kazakhstan, Bureau of National Statistics. Using STEPSsampling.xls, we sampled 60 PSUs out of 266 cities and districts.

(2) Selection of the secondary sampling unit (SSU) - Primary Health Care facilities (PHC)

In each of the 60 selected PSUs, we aimed for 4 PHC facilities, totaling 240 SSUs. Data were taken from The Republican State Enterprise on the Right of Economic Management “Republican Center for Healthcare Development” under the Ministry of Health of the Republic of Kazakhstan. A register of PHC facilities was obtained, including the population served by each. From each selected PSU, using STEPSsampling.xls, 4 SSUs (PHC facilities) were randomly chosen with probability proportional to the population served.

(3) Selection of the tertiary sampling unit (TSU) - households and respondents. Households served as the TSUs. The number of households per PHC facility was calculated as follows:

Household size per PHC facility = 6585 / 240 ≈ 28

Then we calculated final total sample size:

Total Sample Size = 240 × 28 = 6720

A list of households served by the 240 selected PHC facilities was obtained, and households were randomly selected using Randhold.xls. Within each selected household, respondents aged 18–69 were chosen via the Kish methodology, which employs a stratified random selection based on the gender and age of eligible household members [13].

### Data collection

Before the survey, data collection teams received training on interview techniques and physical/biochemical measurements. Interviewers explained the study's goals to each household and obtained verbal informed consent. Face-to-face interviews were conducted, and physical/biochemical measurements were taken on the same day.

### Data variables

A standardized questionnaire, the WHO STEPwise approach to surveillance was employed [14]. In the first stage, information on socio-demographic and behavioral risk factors was collected (age, gender, ethnicity, place of residence, education, occupation, marital status, tobacco use, alcohol consumption, physical activity levels, and fruit and vegetable intake). In the second stage, physical measurements were taken, including weight, height, waist and hip circumference, blood pressure, and heart rate. The third stage involved blood sampling to analyze blood sugar and cholesterol levels.

Smoking status was assessed with 40 questions. A respondent who answered “Yes” to “Do you currently smoke any tobacco products?” was considered a current smoker. Ever smoking was assessed by asking if they had ever smoked any tobacco product. Details on the frequency and quantity of cigarettes, hand-rolled cigarettes, pipes, hookahs, or other products were also collected. We also asked participants about their age at smoking initiation, any quit attempts they had made, and whether they received advice to quit smoking from healthcare providers in the past 12 months.

Smokeless tobacco usage was assessed by asking about snuff, chewing tobacco, moist snuff, or nasvay. Secondhand smoke exposure was determined by asking if anyone had smoked in their home or workplace in the past 30 days.

### Statistical analysis

All data were initially entered into Microsoft Excel and then transferred to SPSS (version 24.0) for detailed analysis. The Kolmogorov-Smirnov test was used to assess data distribution, confirming normality. Mean differences between two groups were analyzed using Student's *t*-test, with results reported as means and 95 % confidence intervals (CI).

To visualize the prevalence of smoking in Kazakhstan's regions, a map was created using survey data via Datawrapper https://www.datawrapper.de/.

## Results

### Demographic characteristics

A total of 6720 participants completed the questionnaire: 50.1 % male and 49.9 % female. The average age of male respondents was 40 years (95 % CI: 39.6–40.4), and that of female respondents was 41 years (95 % CI: 40.6–41.4). Most respondents had higher education (45.7 %) and were officially married (66.2 %). The most common ethnic groups were Kazakhs (65.1 %) and Russians (23.1 %) (Table 2). The regional distribution of participants is presented in Table 1.

**Table 1:** Regional distribution of study participants.

Region	Amount	Percentange, %
Astana city	448	6.7
Almaty city	560	8.3
Akmola region	336	5.0
Aktobe region	336	5.0
Almaty region	560	8.3
Atyrau region	336	5.0
West Kazakhstan region	224	3.3
Zhambyl region	448	6.7
Karaganda region	448	6.7
Kostanay region	336	5.0
Kyzylorda region	336	5.0
Mangystau region	336	5.0
Turkestan region	560	8.3
Pavlodar region	336	5.0
North Kazakhstan region	224	3.3
East Kazakhstan region	448	6.7
Shymkent city	448	6.7
Total	6720	100.0

**Table 2:** Socio-demographic and physical characteristics of survey participants depending on smoking status (*n* = 6720).

Variables	Current smokers (%, 95 % CI)			Non-smokers (%, 95 % CI)		
**Gender**						
Male	79.3 (76.8–81.8)			43.2 (41.5–44.9)		
Female	20.7 (15.8–25.6)			56.7 (55.0–58.4)		
	Male	Female	Total	Male	Female	Total
**Age**						
18–29	23.3 (20.7–26.0)	22.9 (17.8–28.5)	23.2 (21.0–25.4)	27.6 (25.5–29.8)	25.3 (23.3–27.4)	26.3 (24.7–28.0)
30–44	43.0 (40.0–46.0)	48.9 (43.3–54.5)	44.2 (41.5–46.8)	34.6 (32.2–37.0)	31.7 (29.3–34.2)	32.9 (30.8–35.0)
45–59	26.5 (23.9–29.3)	23.7 (19.0–29.0)	25.9 (23.6–28.3)	25.2 (23.0–27.5)	28.3 (26.0–30.7)	27.0 (25.2–28.9)
60–69	7.2 (5.7–8.9)	4.5 (2.3–8.1)	6.6 (5.3–8.1)	12.6 (11.0–14.4)	14.7 (12.7–16.9)	13.8 (12.3–15.3)
**Education**						
No schooling	1.4 (0.7–2.2)	0.4 (0.0–1.1)	1.2 (0.6–1.8)	0.9 (0.5–1.3)	1.1 (0.7–1.5)	1.0 (0.6–1.4)
Completed primary education	0.3 (0.0–0.7)	0.0 (0.0–0.0)	0.2 (0.0–0.5)	0.2 (0.0–0.5)	0.1 (0.0–0.3)	0.1 (0.0–0.3)
Completed secondary education (9 grade)	7.8 (6.1–9.6)	5.3 (2.6–8.0)	7.2 (5.8–8.7)	5.9 (4.9–6.9)	6.0 (5.0–7.0)	5.9 (5.0–6.8)
Completed secondary education (11 grade)	28.0 (25.4–30.7)	31.2 (26.2–36.4)	28.7 (26.4–31.1)	26.2 (24.3–28.2)	26.1 (24.2–28.0)	26.2 (24.7–27.7)
Higher	46.1 (43.1–49.1)	49.6 (44.6–54.6)	46.8 (44.3–49.3)	44.8 (42.5–47.1)	46.0 (43.8–48.2)	45.5 (43.5–47.5)
Master/Doctoral	15.7 (13.5–18.0)	12.4 (8.6–16.7)	15.0 (13.1–16.9)	19.9 (18.0–21.8)	18.9 (17.0–20.8)	19.3 (17.7–20.9)
No answer	0.8 (0.2–1.4)	1.2 (0.0–2.6)	0.4 (0.1–0.8)	2.2 (1.6–2.8)	1.7 (1.1–2.3)	0.6 (0.4–0.9)
**Employment status**						
State employee	11.0 (9.1–13.1)	7.9 (4.6–12.3)	10.4 (8.8–12.2)	12.2 (10.7–13.9)	14.0 (12.3–15.9)	13.2 (12.0–14.5)
Private sector worker	53.8 (50.7–56.9)	50.8 (44.6–57.0)	53.2 (50.5–55.9)	43.1 (40.6–45.7)	28.8 (26.2–31.5)	35.0 (33.2–36.8)
Budget employee	8.2 (6.6–10.1)	9.0 (5.8–13.4)	8.3 (7.0–9.9)	9.7 (8.2–11.4)	15.7 (13.8–17.7)	13.1 (11.7–14.5)
Entrepreneur	10.8 (8.9–13.0)	10.9 (7.5–15.5)	10.8 (9.2–12.7)	10.6 (9.1–12.2)	5.3 (4.0–7.0)	7.6 (6.6–8.8)
Agricultural worker	1.2 (0.6–2.1)	0.4 (0.0–2.1)	1.0 (0.5–1.7)	1.2 (0.8–1.8)	0.3 (0.1–1.0)	0.7 (0.4–1.1)
Student	2.4 (1.5–3.6)	1.5 (0.4–3.7)	2.2 (1.5–3.0)	5.5 (4.6–6.6)	4.3 (3.4–5.5)	4.8 (4.1–5.6)
A housewife	0.0 (0.0–0.0)	13.2 (9.4–17.7)	2.9 (2.0–3.9)	0.0 (0.0–0.0)	14.3 (12.6–16.1)	8.5 (7.5–9.6)
Pensioner	5.0 (3.8–6.6)	3.0 (1.3–5.9)	4.6 (3.6–5.8)	8.8 (7.6–10.1)	12.4 (10.9–14.0)	10.8 (9.8–11.9)
Unemployed (able to work)	6.1 (4.8–7.8)	3.4 (1.5–6.4)	5.4 (4.4–6.6)	7.1 (5.9–8.4)	3.9 (3.0–5.1)	4.9 (4.1–5.8)
Unemployed (unable to work)	0.9 (0.4–1.8)	0.0 (0.0–0.0)	0.7 (0.3–1.4)	1.4 (0.9–2.0)	0.5 (0.3–1.0)	0.9 (0.6–1.3)
No answer	0.7 (0.3–1.4)	0.0 (0.0–0.0)	0.5 (0.2–1.0)	0.4 (0.2–0.8)	0.4 (0.2–0.8)	0.4 (0.3–0.7)
**Marital status**						
Single, not married	22.6 (20.2–25.1)	25.6 (20.7–31.0)	23.2 (21.0–25.5)	25.2 (23.4–27.1)	20.7 (19.2–22.3)	22.6 (21.2–24.1)
Married	69.1 (66.4–71.7)	46.2 (41.0–51.4)	64.3 (61.8–66.7)	70.3 (68.5–72.0)	63.8 (61.9–65.7)	66.6 (65.1–68.2)
Married but living separately	0.5 (0.2–1.2)	2.3 (1.0–4.7)	0.9 (0.5–1.5)	0.6 (0.3–1.0)	0.9 (0.6–1.4)	0.8 (0.5–1.1)
Divorced	5.0 (3.7–6.5)	15.8 (12.0–20.1)	7.2 (5.8–8.7)	2.5 (1.8–3.2)	7.6 (6.5–8.9)	5.4 (4.6–6.3)
Widower/widow	0.7 (0.3–1.4)	6.0 (3.8–8.8)	1.8 (1.1–2.6)	0.7 (0.4–1.1)	6.0 (5.1–7.0)	3.7 (3.1–4.4)
Civil marriage	1.8 (1.1–2.8)	4.1 (2.3–6.7)	2.3 (1.5–3.2)	0.6 (0.3–1.0)	0.9 (0.6–1.4)	0.8 (0.5–1.1)
No answer	0.4 (0.1–1.0)	0.0 (0.0–0.0)	0.3 (0.1–0.8)	0.1 (0.0–0.3)	0.1 (0.0–0.3)	0.1 (0.0–0.3)
**Ethnicity**						
Kazakh	65.5 (63.3–67.6)	37.6 (31.7–43.7)	59.7 (58.0–61.4)	68.3 (66.6–70.0)	64.9 (63.2–66.6)	66.4 (65.1–67.8)
Russian	22.6 (20.4–24.9)	54.5 (49.9–59.1)	29.2 (27.5–31.0)	19.4 (17.7–21.3)	23.3 (21.8–24.9)	21.6 (20.4–22.9)
Uzbek	2.1 (1.3–3.2)	0.0 (0.0–0.0)	1.6 (1.0–2.4)	3.5 (2.8–4.3)	3.2 (2.7–3.9)	3.3 (2.8–3.9)
Ukranian	1.3 (0.7–2.3)	1.5 (0.5–3.8)	1.3 (0.8–2.1)	2.0 (1.5–2.6)	1.5 (1.0–2.1)	1.7 (1.3–2.3)
Uigur	0.8 (0.3–1.6)	0.4 (0.0–2.1)	0.7 (0.3–1.3)	0.5 (0.3–0.8)	0.5 (0.3–0.8)	0.5 (0.4–0.9)
Tatar	1.8 (1.1–2.9)	1.5 (0.4–3.8)	1.7 (1.1–2.6)	1.5 (1.1–2.1)	1.9 (1.5–2.5)	1.7 (1.3–2.2)
Other	6.0 (4.7–7.5)	4.5 (2.3–7.9)	5.7 (4.6–6.9)	4.7 (3.9–5.6)	4.8 (4.1–5.6)	4.7 (4.1–5.4)
**BMI groups**						
<18.5	1.2 (0.6–2.0)	3.3 (1.6–6.0)	1.7 (1.0–2.6)	1.8 (1.3–2.4)	5.4 (4.6–6.3)	3.9 (3.3–4.5)
18.5–24.9	37.3 (34.8–39.8)	42.0 (37.1–47.0)	38.3 (36.3–40.3)	34.8 (32.7–36.9)	42.9 (41.0–44.9)	39.4 (37.8–41.0)
25–29.9	39.9 (37.4–42.4)	28.2 (23.1–33.8)	37.5 (35.5–39.5)	44.3 (42.2–46.5)	29.7 (27.5–32.0)	36.1 (34.5–37.7)
>30	21.4 (19.2–23.7)	26.3 (21.3–31.8)	22.4 (20.6–24.3)	18.9 (17.3–20.5)	21.8 (20.0–23.7)	20.6 (19.2–22.0)
**Arterial pressure, mmHg (mean, 95 % CI)**						
Systolic arterial pressure	124.9 (123.9–125.8)	117.6 (115.5–119.7)	123.4 (122.5–124.3)	124.2 (123.2–124.6)	118.5 (117.8–119.1)	121 (120.5–121.5)
Diastolic arterial pressure	82.1 (81.5–82.8)	79.9 (78.5–81.5)	81.7 (81.1–82.3	81.6 (81.2–82.1)	78.6 (78.1–79.0)	79.9 (79.6–80.2)
**Heart rate, bpm**	74.6 (73.9–75.2)	74.4 (73.3–75.5)	74.5 (73.9–75.1)	73.5 (73.1–73.9)	73.7 (73.4–74.1)	73.6 (73.4–73.9)

### Smoking prevalence

**Fig. 1:**
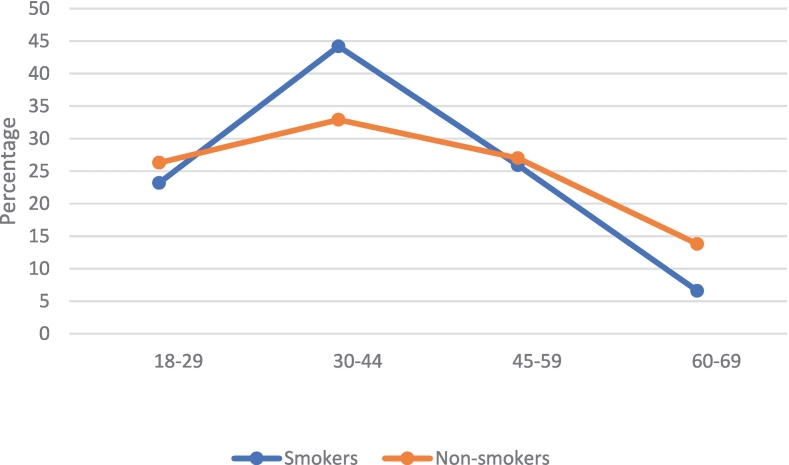
Age group distribution of smokers and non-smokers.

Overall, 19.1 % of respondents were current smokers. Smoking was more common among men (30.3 %) than women (7.9 %). By age group, respondents aged 30–44 had a higher proportion of smokers (44.2 %) compared to non-smokers (32.9 %) (Fig. 1). Among smokers, private-sector employees were more common compared to non-smokers (53.2 % vs. 35.0 %). Education level and marital status did not differ substantially between smokers and non-smokers. No notable differences were observed between smoking status (current vs. non-smoker) and BMI, blood pressure, or heart rate.

The most commonly smoked tobacco product was manufactured cigarettes, reported by 1150 respondents (17.1 %). Additionally, 97 respondents (1.4 %) used heated tobacco products (HTPs), and 32 respondents (0.5 %) reported smoking a waterpipe (hookah). Hand-rolled cigarettes and pipes were less common, each at 0.1 %.

### Regional variations

**Fig. 2:**
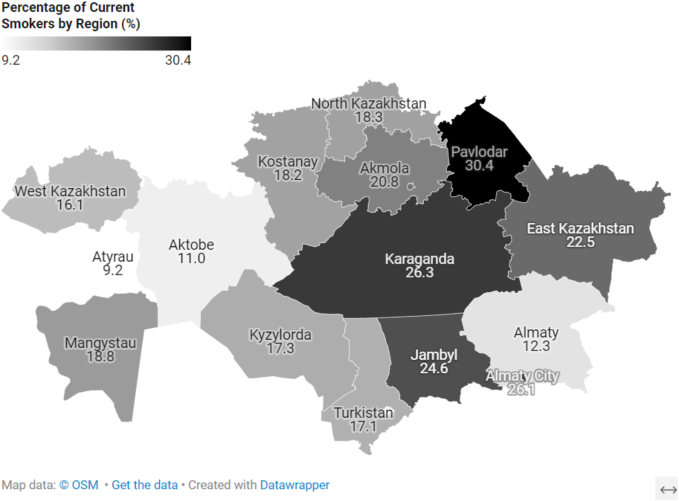
Distribution of current smokers by region of Kazakhstan.

**Fig. 3:**
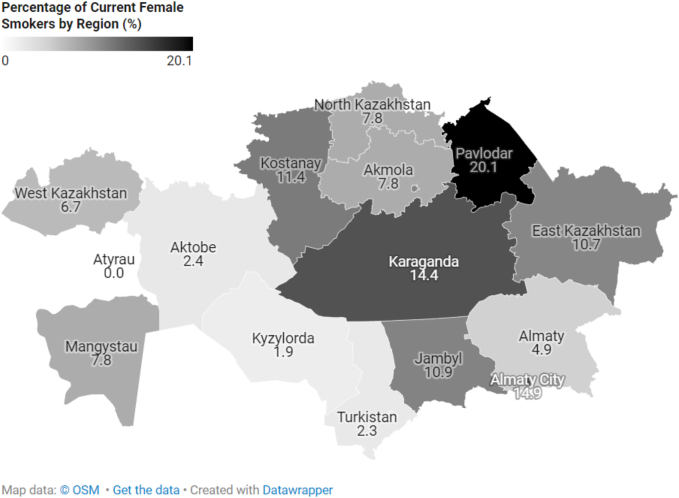
Distribution of Current Female Smokers by Region of Kazakhstan.

**Fig. 4:**
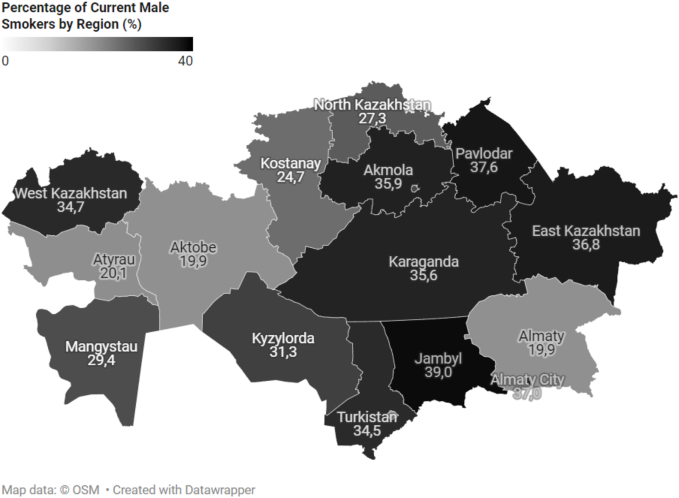
Distribution of Current Male Smokers by Region of Kazakhstan.

By region, the Atyrau region had the lowest smoking prevalence (9.2 %), while the Pavlodar region had the highest (30.4 %) (Fig. 2). Fig. 3, Fig. 4 illustrate the regional prevalence of current female and male smokers, respectively. Among women, the highest prevalence was in Pavlodar (20.1 %) and the lowest in Atyrau (0 %). Among men, the highest prevalence was also in Pavlodar (37.6 %), while the lowest was in Shymkent (18.1 %).

### Smoking behavior-cessation attempts

Of the 1284 current smokers, 1144 (89.1 %) smoked daily. Among them, 926 were men (91.0 % of male smokers), and 218 were women (82.0 % of female smokers) (Table 3). The average age of smoking initiation was 19.2 years (95 % CI: 18.9–19.5), with a range of 5–60 years.

**Table 3:** Characteristics of smoking respondents.

Variables		Male (%, 95 % CI)	Female (%, 95 % CI)
**Daily smokers of any tobacco**	Yes	91.0 (89.3–92.5)	82.0 (77.6–85.8)
	No	9.0 (7.5–10.7)	18.0 (14.2–22.4)
**Number of different tobacco products smoked daily** (mean, 95 % CI)			
number of manufactured cigarettes smoked daily		12.0 (11.4–12.6)	8.5 (7.8–9.2)
number of hand-rolled cigarettes smoked daily		7.6 (6.1–9.1)	10
number of pipes filled with tobacco smoked daily		4.3 (3.1–5.5)	–
number of hookahs sessions (once charged hookah), smoked daily		2 (1.2–2.8)	–
number of other tobacco products smoked daily		8.8 (7.3–10.3)	8.1 (6.6–9.6)

The average number of manufactured cigarettes smoked per day was 11.4 (95 % CI: 10.9–11.9), with men smoking an average of 12.0 per day (95 % CI: 11.5–12.5) and women 8.5 per day (95 % CI: 7.8–9.2). The use of other tobacco products varied. On average, smokers consumed 8.0 hand-rolled cigarettes per day (95 % CI: 6.3–9.7), 4.3 pipes per day (95 % CI: 3.1–5.5), 1.9 hookah sessions per day (95 % CI: 1.2–2.6), and 8.6 other tobacco products per day (95 % CI: 7.1–10.1).

Efforts to quit smoking were reported by a substantial proportion of smokers, with 42.8 % attempting to quit within the past 12 months (Table 4). Among those who had visited a doctor or other healthcare professional in the same period, a larger proportion (58.1 %) did not receive advice to quit smoking, while 41.9 % were advised to quit. Regarding secondhand smoke exposure, 22.3 % of men and 26.8 % of women reported that someone had smoked inside their home in the past 30 days. Additionally, 30.2 % of men and 14.4 % of women stated that they had been exposed to smoking in their workplace during the same period.

**Table 4:** Characteristics of smoking respondents regarding smoking cessation and secondhand smoke.

Variables	Male (%, 95 % CI)	Female (%, 95 % CI)	Total (%, 95 % CI)
**Smokers who have tried to quit smoking in the last 12 months**	43.2 (40.1–46.2)	41.0 (35.1–46.9)	42.8 (40.0–45.4)
**Smokers who saw a healthcare professional in the past 12 months and were advised to quit smoking,**			
yes	37.4 (34.4–40.4)	33.8 (28.1–39.5)	41.9 (34.0–39.3)
no	49.4 (46.3–52.4)	56.4 (50.4–62.4)	58.1 (48.1–53.6)
**Have not seen a health worker within 12 months**	13.2 (11.1–15.2)	9.8 (6.2–13.3)	12.5 (10.6–14.3)
**«Has anyone in your home smoked in the last 30 days? »**			
yes	22.3 (20.9–23.8)	26.8 (25.3–28.3)	24.5 (23.5–25.6)
no	77.7 (76.2–79.1)	73.2 (71.7–74.7)	75.5 (74.4–76.5)
**«In the past 30 days, has anyone smoked in the area where you work? »**			
yes	30.2 (28.6–31.8)	14.4 (13.2–15.6)	22.3 (21.3–23.3)
no	65.4 (63.8–67.0)	82.1 (80.7–83.3)	73.8 (72.7–74.8)
I do not work indoors	4.4 (3.7–5.1)	3.5 (2.9–4.2)	3.9 (3.5–4.4)
**«Have you smoked tobacco products in the past?»**	17.2 (16.0–18.5)	6.7 (5.9–7.6)	11.9 (11.2–12.8)
**Average age of respondents who quit smoking (mean, 95 % CI)**	33.6 (31.9–35.3)	28.8 (27.1–30.5)	31.7 (30.4–33.0)

### Use of smokeless Tobacco

Only 65 respondents (1.0 %) currently used smokeless tobacco products (moist snuff, chewing tobacco, or nasvay), including 46 men (0.7 %) and 19 women (0.3 %).

### Former smokers

A total of 805 respondents (12.0 %) had smoked in the past, including 580 men (17.2 %) and 225 women (6.7 %). The average age of quitting smoking was 32.3 years (95 % CI: 31.1–33.5), with men quitting at 33.6 years (95 % CI: 31.9–35.3) and women at 28.8 years (95 % CI: 27.1–30.5).

## Discussion

The results of this extensive cross-sectional study underscore the widespread prevalence of smoking among the adult population of Kazakhstan and highlight differences in smoking behaviors among various demographic groups, emphasizing the need for effective government measures to reduce smoking rates.

According to our data, the prevalence of smoking in Kazakhstan was 19.1 %, which is slightly lower than the rates obtained in the GATS studies in 2014 and 2019, at 22.9 % and 21.5 %, respectively [10,11]. According to our findings, the prevalence of smoking in Kazakhstan is lower than in Bulgaria (37 %) but higher than in Sweden (9.3 %) [15,16].

Currently, there is a limited amount of data on the prevalence of smoking in other Central Asian countries (Kyrgyzstan, Tajikistan, Turkmenistan, and Uzbekistan), which makes thorough and accurate cross-country comparisons challenging. However, given the available information, a comparison with Uzbekistan appears to be the most logical step. Our data show that the overall prevalence of smoking among respondents in Kazakhstan was 19.1 %, which is comparable to Uzbekistan, where the prevalence of smoking was 19.6 %. However, in our study, the gender difference is much more pronounced: 30.3 % of men and 7.9 % of women smoke. This is considerably higher than the smoking prevalence among women in Uzbekistan, where only 1.1 % reported smoking [17].

Regarding the use of smokeless tobacco products, Kazakhstan shows a much lower level of consumption, with only 1 % of respondents reporting use. In contrast, the use of nasvay in Uzbekistan is much more widespread, with 22.3 % of men using it. These findings highlight notable differences in tobacco consumption patterns between the two countries, particularly in terms of gender disparities and types of tobacco products used.

The prevalence of smoking among men (30.3 %) is higher than among women (7.9 %), which roughly corresponds to the global prevalence – 32.6 % among men and 6.5 % among women [1]. Such gender differences in smoking rates are a common trend and can be influenced by a complex interplay of historical social norms, traditional gender roles, and perceptions of the benefits and costs of smoking [18,19]. Understanding these factors is crucial for developing targeted public health interventions and policies aimed at reducing smoking prevalence and addressing gender-specific needs and challenges.

The highest prevalence of smoking was observed among men aged 30–44 years (44.2 %), which can be explained by social and cultural norms; smoking is perceived as part of the image of a masculine man. Men in this age range are often at the peak of their careers, which may be accompanied by increased stress and pressure. Often, smoking is used as a means of managing tension at work or as a way to take a short break during the workday.

Furthermore, we noted the highest level of smoking among private sector workers (53.2 %), which may be due to the high level of stress due to competitive conditions, as well as less regulation and control of smoking compared to the public sector.

There are notable differences in smoking prevalence across Kazakhstan's regions, ranging from 9.2 % in Atyrau to 30.4 % in Pavlodar. These findings underscore the need for region-specific strategies to effectively reduce smoking rates. Economic factors appear to be a key influence: according to the Bureau of National Statistics, the oil-producing Atyrau region has the highest gross regional product per capita (9.7 million tenge in 2023), whereas Pavlodar's figure is only 2.5 million tenge [20]. Employment patterns also vary between these regions. In Atyrau, government and budgetary employees predominate, while in Pavlodar, private sector workers are more common, helping to explain the observed disparities in smoking prevalence.

We compared our findings on various types of smoked tobacco with data from GATS 2014 and 2019, focusing on manufactured cigarettes, hand-rolled cigarettes, waterpipe (hookah), and HTPs. In our sample, 19.1 % of respondents were current smokers, which closely aligns with the GATS 2019 rate of 20.4 %. Manufactured cigarettes were the most common form of tobacco use (17.1 %), lower than both the 22.2 % reported in GATS 2014 and 19.8 % in GATS 2019. By contrast, HTP use rose slightly to 1.4 %, compared to 1.0 % in GATS 2019, indicating growing interest in these products. Waterpipe smoking declined to 0.5 %, down from 2.9 % in GATS 2014 and 1.2 % in 2019, while hand-rolled cigarette and pipe smoking remained rare at 0.1 %. Overall, our results mirror national patterns but highlight a shift toward alternative tobacco products, particularly HTPs [10,11].

Although there is no safe level of nicotine consumption [21,22], the risk of smoking-related diseases increases with greater daily cigarette use [[23], [24], [25]]. Among current smokers, 89.1 % smoked daily, averaging 11.8 cigarettes per day (95 % CI: 11.4–12.2). This figure is higher than England's 10.7 in 2023 but lower than Iran's 14.5 and Kazakhstan's 15.4 in 2019 [10,11,26,27]. The high proportion of daily smokers and the large number of cigarettes smoked per day indicate a high level of nicotine dependence among smokers in Kazakhstan.

Alarmingly, only 36.7 % of current smokers who interacted with healthcare workers in the past year reported being advised to quit smoking, compared to 53 % in the USA [28]. Nevertheless, 42.8 % made a quit attempt in the past 12 months, which is higher than 30 % in Kazakhstan in 2019 [11] but lower than 51.3 % in the USA for 2018–2019 [29]. These findings highlight the low frequency of physician-initiated smoking cessation discussions in Kazakhstan.

Regarding passive smoking, more women (26.8 %) were exposed at home compared to men (22.3 %), reflecting a similar pattern reported in India [30]. However, men faced higher exposure in the workplace (30.2 % vs. 14.4 %). These rates exceed 2019 levels in Kazakhstan (14.6 % for men and 7.8 % for women). Such demographic patterns may be tied to gender roles in the workplace and at home.

Smokeless tobacco products are not popular in Kazakhstan, with only 1 % of respondents using them. Globally, this varies from 1.1 % in Thailand to 51.4 % in Myanmar [31].

While quitting smoking benefits health at any age, stopping by age 35 yields a mortality rate similar to never smokers [32]. In our study, the average age of quitting was 32.3 years (95 % CI: 31.1–33.5)—within the global range of 25–44 years [33]. Women tended to quit earlier (28.8 years, 95 % CI: 27.1–30.5) than men (33.6 years, 95 % CI: 31.9–35.3), which is comparable to Eastern Europe (26–27 years) [34] and may correlate with pregnancy and childbearing decisions.

Our study covers all regions of Kazakhstan using a weighted multistage cluster sampling method, expanding upon previous GATS data that provided national but not detailed regional insights. By including all 17 regions, this study identifies substantial disparities in smoking prevalence, offering valuable data for policymakers and health officials to tailor region-specific interventions.

These findings support a multi-faceted approach to tobacco control, including stricter enforcement of smoking bans, increased public health campaigns tailored to different demographics and regions, and more robust support from healthcare providers to encourage smoking cessation. Strengthening tobacco taxes and regulations on advertising and sales can also help reduce smoking rates.

Ultimately, our research provides a foundation for future policy and research, emphasizing the need for tailored interventions to address both cultural norms and structural challenges in Kazakhstan.

## Limitations

The cross-sectional nature of the study limits the ability to establish cause-and-effect relationships between variables. We can only speak of associations, not causality. Despite a high participation rate (95 %), there is potential for systematic bias if those who declined participation have different smoking behaviors. The study coincided with the COVID-19 pandemic (2021−2022), potentially affecting smoking habits, healthcare access, and willingness to participate.

## Conclusion

This study revealed a high prevalence of smoking, particularly among men, among the representative population in Kazakhstan. A low percentage of smoking cessation recommendations received by smokers from medical personnel was found. Besides, a high level of passive smoking both at home and at work was identified. This study provides a thorough view of the prevalence of smoking in Kazakhstan, identifying differences between individual population groups. The results lay a solid foundation for future research and can be used to develop anti-smoking policy.

## Funding

The study was supported by the grant of the Ministry of Healthcare of the Republic of Kazakhstan “Epigenetics and prevention of non-communicable diseases in Kazakhstan: a personalized approach and biological age prediction” (Grant Number BR27304987).

## CRediT authorship contribution statement

**Anel Ibrayeva:** Writing – review & editing, Writing – original draft, Project administration, Methodology, Investigation, Formal analysis, Data curation, Conceptualization. **Marat Shoranov:** Writing – review & editing, Writing – original draft, Supervision, Software, Resources, Methodology, Investigation, Data curation, Conceptualization. **Rassulbek Aipov:** Writing – review & editing, Writing – original draft, Investigation, Formal analysis. **Adil Katarbayev:** Writing – review & editing, Writing – original draft, Visualization, Software, Investigation. **Shynar Tanabayeva:** Writing – review & editing, Writing – original draft, Methodology, Investigation. **Ildar Fakhradiyev:** Writing – review & editing, Writing – original draft, Visualization, Validation, Methodology, Investigation, Formal analysis.

## Declaration of competing interest

The authors declare that they have no known competing financial interests or personal relationships that could have appeared to influence the work reported in this paper.
